# COVID-19 vaccine and booster hesitation around the world: A literature review

**DOI:** 10.3389/fmed.2022.1054557

**Published:** 2023-01-12

**Authors:** Aashka Shah, Olivia C. Coiado

**Affiliations:** ^1^Carle Illinois College of Medicine, University of Illinois at Urbana-Champaign, Urbana, IL, United States; ^2^Department of Biomedical and Translational Sciences, University of Illinois at Urbana-Champaign, Urbana, IL, United States

**Keywords:** COVID-19, vaccine hesitancy, vaccination education, COVID-19 booster vaccine, global vaccine literacy, COVID-19 vaccination rate, COVID-19 booster rates

## Abstract

The development of COVID-19 vaccines has helped limit the extent of the pandemic, which over the past 2 years has claimed the lived of millions of people. The Moderna and Pfizer COVID-19 vaccines were the first to be manufactured using mRNA technology. Since then, other manufacturers have built their own vaccines which utilize adenovirus vector, whole inactivated coronavirus, and protein subunit methods. Given the continued mutation of the SARS-CoV-2 virus, a booster of the COVID-19 vaccine offers additional protection for citizens, especially those with comorbid conditions. However, uptake of the vaccine and booster has faced hurdles. This literature review aims to analyze the acceptance of the COVID-19 booster among different populations throughout the world. Keywords searched include “COVID-19 vaccine rates OR COVID-19 booster rates,” “COVID-19 vaccine hesitancy,” “COVID-19 booster hesitancy,” “reasons against COVID-19 vaccine,” “reasons for COVID-19 vaccine,” and “COVID-19 vaccine acceptance” (for each country). Research articles indexed in PubMed, University of Illinois Urbana-Champaign Library, and Google Scholar were included. Despite the proven effectiveness of the COVID-19 booster, vaccine hesitancy is still causing suboptimal compliance to the primary vaccine and booster, thus slowing down control of the pandemic. Reasons for vaccine hesitancy differ by country and acceptance is affected by misinformation, political circumstances, and cultural values. Among the most common reasons found are distrust in the government, a lack of safety information, and fear of side effects. Uptake of the COVID-19 vaccine has also been delayed in low and middle income countries due to resource allocation and as a result, these countries have fallen behind vaccination benchmarks. The future of COVID-19 vaccination is unknown, but vaccine mandates and additional booster doses are a possibility. Determining the ethical impact that these policies could have will allow for the best implementation.

## 1. Introduction

Since January 23rd 2020, the CDC has reported a total of 86,600,000 SARS-CoV-2 (COVID-19) cases in the US, with a death count totaling 1,010,000 (1). Elderly patients and patients with pre-existing chronic conditions (heart failure, obesity, diabetes, liver cirrhosis, chronic kidney disease, cancer, and transplanted organs) have an increased risk of poor COVID-19 outcomes (2). With the increase in mortality rates for these high-risk patients, a COVID-19 vaccine was necessary to decrease the dire outcomes of the virus. On December 11th of 2020, less than a year after the start of the pandemic, an emergency use authorization was approved by the Food and Drug Administration (FDA) for the Pfizer, BioNTech COVID-19 vaccine in the US (3). Since then, three other vaccines have been authorized by the FDA including Moderna, Janssen (Johnson & Johnson), and the Novavax bivalent vaccine. Currently, there are over 10 approved vaccines around the world, each of which work by using mRNA, adenovirus vector, whole inactivated coronavirus, or protein subunit mechanisms (4). All of the approved vaccines have a primary regimen of two doses for optimal efficacy (with the exception of the Johnson & Johnson vaccine which uses a one dose primary regimen). While distribution of the vaccine depends on the country, in total, 68.5% of the world's population has received at least one dose of an approved vaccine (5). These vaccines offered a chance to control the pandemic.

Even with gradually increasing vaccination rates, SARS-CoV-2 has continued to mutate, posing a challenge in containing the pandemic. So far, there have been five major strains of SARS-CoV-2: the Alpha, Beta, Gamma, Delta, and Omicron variants (6). These mutants occur because of a modification to the outer protein on the virus, the spike protein- a surface protein which allows the virus to penetrate host cells. Mutations which lead to a change in conformation of the spike protein could allow the virus to escape detection by immune systems (7, 8). While these mutations pose an additional obstacle in efforts to contain the pandemic, studies have shown that the COVID-19 vaccines are effective at preventing serious illness even in patients infected with the mutant strains (9). However, like any other vaccine, antibody levels from the COVID-19 vaccine fall over time (8, 10). Within 6 months of receiving the complete vaccine course, antibodies to SARS-CoV-2 were found to be substantially decreased, especially in the immunocompromised and elderly populations (11–13). To ensure adequate protection against hospitalization and serious disease, especially in the face of the mutated strains, health organizations around the world have recommended the implementation of booster vaccinations.

Initially, during Phase III clinical trials, 2-dose mRNA COVID-19 vaccines had published effective rates ranging from 78 to 95%, depending on the vaccine developer (10). In the US, only Pfizer (BNT162b2) and Moderna (mRNA-1273) vaccines are available. These vaccines initially showed an effective rate of 94–95% but mutations have changed these values (14). As the Delta variant became more prominent in fall of 2021, the average vaccine effectiveness, 180 days from the last vaccine dose, declined to 76% and then to 34% with the Omicron variant (15, 16). This decrease in vaccination effectiveness can be attributed to natural decreases in antibody titers after vaccination and the gradual accumulation of mutations in SARS-CoV-2 spike proteins per variant (17, 18). The initial optimism of the vaccine faded away as cases rose with variants and hospitals filled up once more with COVID-19 patients. While vaccines have proven to be effective in minimizing disease progression, continued surges in COVID-19 cases have overwhelmed hospitals resources. In an effort to decrease COVID-19 recurrence in communities and ease the ongoing tension on hospitals across the US, the CDC recommends that all those who have received a mRNA vaccine more than 5 months ago, or have received an adenovirus based vaccine more than 2 months ago, also receive the third booster and people ages 5 years or older receive the updated bivalent booster 2 months after their last dose (19, 20). With mutations of SARS-CoV-2, booster vaccinations have become important in maintaining immunity in the general population. Initial studies demonstrate that boosters have been effective in decreasing hospitalizations and emergency room visits due to COVID-19, providing a general decrease in viral transmissibility, and shortening recovery time due to improved immunity (21, 22). Thus, the ability of the booster to prevent hospitalization is a major benefit in ensuring that these patients have favorable outcomes in the event they are infected.

However, given how recent the implementation of booster doses has been, studies regarding the uptake of COVID-19 boosters are limited. Acceptance of vaccines and their boosters have not been universal, and this may have far reaching consequences for the future of the pandemic. This paper aims to review COVID-19 vaccine and booster hesitancy throughout America and worldwide. We discuss the knowledge surrounding COVID-19 and its correlation with vaccine acceptance rate as well as the ethical aspects of the booster and mandatory vaccination.

## 2. Research methods

This analysis examines and reviews literature from 2020 to 2022 involving the public response to the vaccine and booster and the proposed future of the vaccine. Multiple research search engines of PubMed, University of Illinois Urbana-Champaign Library, and Google Scholar were used. While compiling literature for this review, several methodologies were followed.

For review of COVID-19 vaccine and booster hesitancy, “COVID-19 vaccine rates OR COVID-19 booster rates,” “COVID-19 vaccine hesitancy,” and “COVID-19 booster hesitancy” were searched. To summarize reasons of vaccine hesitancy by country and region, “reasons against COVID-19 vaccine,” “reasons for COVID-19 vaccine,” and “COVID-19 vaccine acceptance” were searched for each country that was analyzed. Results were filtered by article type to examine meta-analyzes, literature reviews, systematic reviews, reviews, clinical trials, and randomized control trials. Literature was then included based on direct relevance to reasons of COVID-19 vaccine or booster hesitancy. Published journal papers were primarily used as the main form of article type as up-to-date literature was the goal of the study.

In addition to the compiled list of literature above, researchers conducted directed searches on topics relating to past vaccination campaigns, prior pandemics, and the method of vaccine rollout in different areas of the world. Special attention was given to different factors which could have affected each country's response to the pandemic and vaccine.

Multiple search engines were used to reduce any possible bias by omission. As mentioned above, PubMed, University of Illinois Urbana-Champaign Library, and Google Scholar were all equally used. PubMed was the primary search engine for our research. Furthermore, minimization of search criteria allowed for inclusion of all relevant papers.

## 3. Booster acceptance and hesitancy in America

On average, COVID-19 booster uptake in the US was ~43.99%, with the rest being booster hesitant (5). A few factors leading to booster hesitancy in the US could be low vaccine literacy, concern of side effects, and mistrust in the government/big pharmaceutical companies.

Based on the concept of health literacy, vaccine literacy refers to the ability of the individual to understand the health implications that the vaccine provides as well as be aware of resources which can guide them in making decisions regarding the vaccine (23, 24). Low levels of vaccine literacy are associated with a decreased desire to partake in preventative measures (like vaccines). Vaccine literacy is scored on three different subsections. In each subsection, decreased vaccine literacy is associated with increased vaccine hesitancy. Patients who had difficulty in understanding COVID-19 information less frequently engaged in preventative measures (like mask-wearing and hand washing). Those who had trouble accessing sufficient information about the vaccine were also more likely to experience vaccine hesitancy (25). This demonstrates the importance of public health education to increase vaccine uptake as they are seen to be very strongly correlated (25).

Misinformation about the COVID-19 vaccine is an obstacle to vaccine literacy and is a large reason why Americans choose to not be vaccinated (26). It continues to be a driver in the low acceptance rate of the booster. Fact checking and refuting false claims is not enough to counteract misinformation. Rather, to combat the misinformation in the media, individual conversations with a health professional and personal anecdotes have a greater effect in conveying a message (27). Administration of the COVID-19 vaccine series was primarily done in large centers. For the booster shot, and subsequent COVID-19 shots, administering them in a clinic setting will allow patients to have conversations with a trusted physician and allow for the COVID-19 booster to become a part of their regular appointment (28).

Active learning strategies are the most effective method for health education compared to passive reading (29). By invoking strategies such as the “ask-tell-ask” method, physicians can contribute to minimizing the extent that mis-informed narratives have on the decision-making process instead of relying on patients to read brochures and literature. Furthermore, implementing these strategies will also play a role in increasing health literacy and decreasing the effect that false information plays on the vaccine campaign.

Personal experience is a major factor in decision making and the role of anecdotal evidence has been proven to be a large aspect of decision making (30, 31). This has also proved true in the case of the COVID-19 vaccine, especially with the booster. Experiences from the first two doses play into the decision to get the booster dose (28). The most common side effects from the COVID-19 vaccine are fatigue and injection site tenderness (32). In the US, like many countries, concerns of vaccine side effects play into vaccine hesitancy. Though there have been no studies done on the association of booster side effects compared to the original doses, it can be assumed that side effects that do develop are similar between doses. Studies done on reactogenicity of the different vaccines found that heterologous boosting was associated with greater symptoms but that all vaccine combinations showed an acceptable minimal side effect profile (33). Though stories of severe side effects invoke an emotional response and thus stay engrained in memories, greater media attention to the conclusions of controlled scientific studies will emphasize the rare occurrence of severe side effects.

Social media was a unique challenge during the COVID-19 era, and this pandemic is the first major health crisis to be affected on a large scale by social media, 75–80% of Americans look to the internet for health information, often through social media (34). This is relevant to people who are pro-vaccine as well as vaccine hesitant. Betsch et al., found that even a 5–10 min exposure to vaccine critical content leads to increased perceived risk of vaccination and decreases the intention to vaccinate (35). Reasons why social media play such a large role in vaccine perception are multifactorial. One major factor is due to the vivid narrative and imagery that social media is able to create (36). Personal experiences with the first COVID-19 vaccine can be shared on social media and those with negative outcomes (ex: side effects, long term effects, long wait times, etc.) will have a stronger and lasting effect on readers than positive stories. Choosing to share stories and posts that emphasize the negative outcomes of the COVID-19 vaccine creates an over-emphasis of the rare side effects and social media users are skewed in their perception of the occurrences of these negative outcomes (37). Furthermore, it is often difficult for users to determine the scientific accuracy of the postings on social media. Many posts contain no scientific backing and anti-vaxxers will select certain sentences from a study which reinforce their view and fail to summarize a study in its entirety (37). With the contribution of social media in the public perception of vaccinations, social media dissemination of vaccine adverse events results in epidemics that last 150% longer (38). This may be seen during the COVID-19 pandemic as well, as people hesitate to get the booster due to negative perceptions of the vaccine created by stories and posts on social media.

A study showed that 13% of participants reported that they were unsure about whether they would be willing to receive the booster or not while 87% had made a strong decision (39). While the latter may not be able to have their opinion altered, the former may be persuaded either way. Public health education campaigns and effective media tactics play a large role in pushing the uncertain population to one side or another. Mass media messages have limited benefit for a vaccine campaign and to enhance their effect, the message should be tailored to the altruism of receiving the vaccine, emphasis on the dangers of COVID-19, and the regret of not having received the COVID-19 vaccine and booster. However, more effective than a media campaign is the ability to converse with a health professional as mentioned above. This method proves the most effective in increasing turn-over (40). Family doctors and other physicians should take the time to explain the benefits of receiving the COVID-19 booster as this could have a lasting impression on the opinion of the patient.

With the number of changing updates that have surrounded the COVID-19 pandemic, citizens feel uncertain in their confidence in the US government to make decisions in the best interest of the public (41). Statements regarding the response to the COVID-19 pandemic in America such as “The government provided protection to the most vulnerable populations” and “The government clearly communicated to everyone on the best practices to protect themselves” received low scores relative to other countries. When the vaccine was first rolled out, a booster shot was not anticipated. Furthermore, much of the media promised for the end of the pandemic if people received the vaccine. A year after the vaccine was approved, the US government is now encouraging patients to receive a third vaccine. This can promote feelings of confusion and uncertainty regarding what aspects to trust the government and scientific community. Among those who are unwilling to receive the vaccine, 75% of Americans are not confident that the COVID-19 vaccines were properly tested for safety and effectiveness (42). Moreover, as politics play into the administration of the booster- trust in the US government is decreasing even further. In August of 2021, when news of the booster first gained traction, the US government dismissed the booster as a money making scheme for pharmaceutical companies (43). This reflects a sentiment that people share about corporations and political figures, thus reaffirming the thought leads to increased vaccine unwillingness.

The initial campaign for the COVID-19 vaccine in America is very similar to that of the booster. To raise vaccination rates, companies began to offer free incentives for those who got vaccinated. Interestingly, for certain ethnic groups such as Blacks and Latino Americans, monetary incentives to receive the booster have decreased the trust in the vaccine while for the general population, vaccine acceptance modestly increased with monetary incentives (44). This may be attributed to historical accounts where Blacks were exploited and ill-treated by the medical field (44). Monetary incentives may feel like a pay-off and increase suspicion for the safety and benefit of the vaccine. With the booster, public health officials have another chance to methodize how to increase the booster rate. With the lessons learned from the marketing of the vaccine, officials can make sure to market the booster in audience specific ways as we have seen that trends that hold true for one group may not for another.

**Figure 1:**
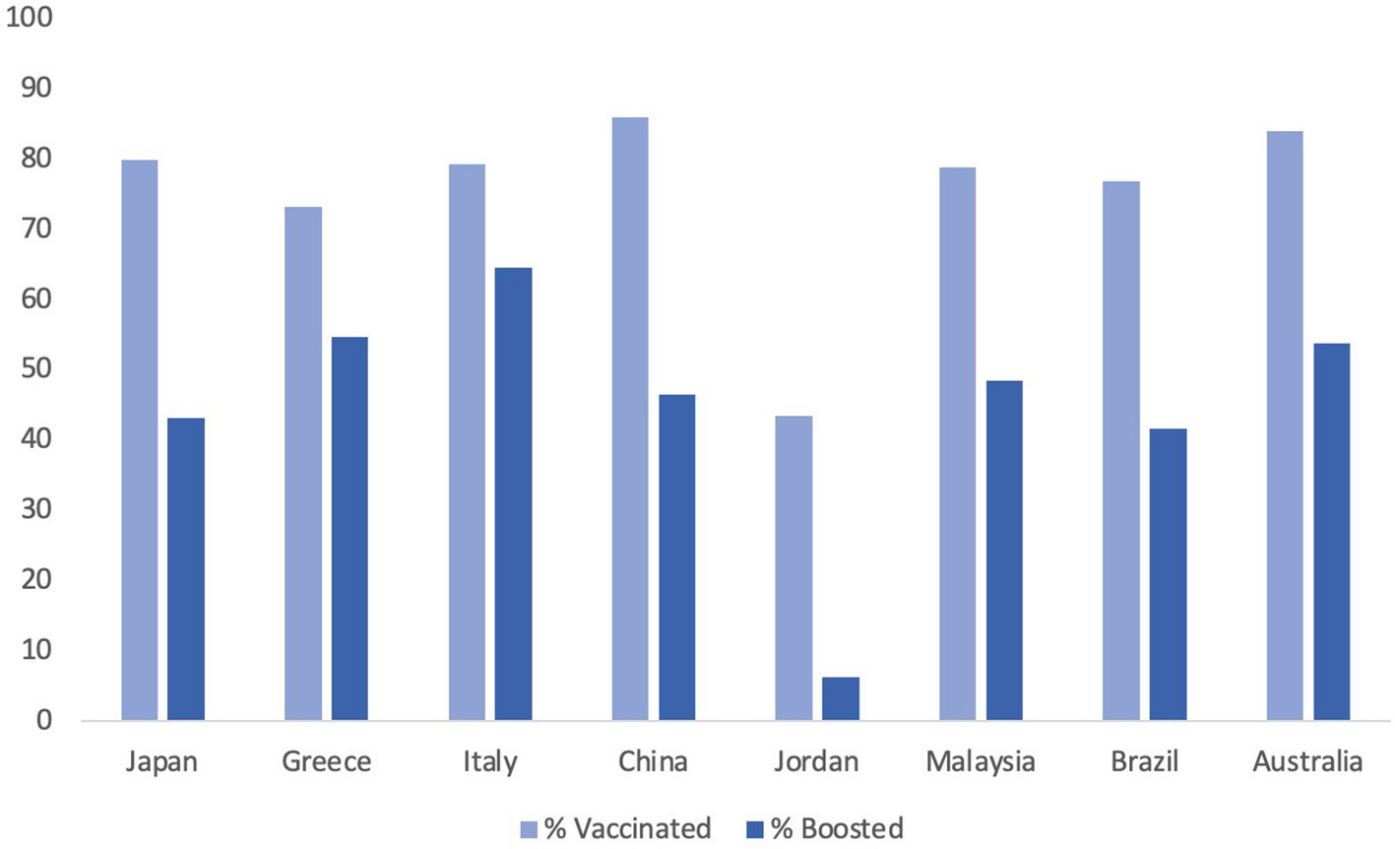
A country specific visualization of COVID-19 vaccination and booster rates by December 2022.

The attitude of healthcare workers in the US toward the booster dose also plays a role in the acceptance in the general public. In a study directed toward healthcare workers, it was previously found that only 1/3rd of healthcare workers was ready to take the COVID-19 vaccine as soon as it was released. Figure 1 demonstrates COVID-19 vaccination rates in different countries around the world. Down the line, those statistics have improved as the vaccine has been approved for longer. Only ~8% of healthcare workers state that they would be unwilling to take the vaccine 2 months later (9, 45). Among both groups, vaccinated and unvaccinated, 2/3rds of participants stated that it was probable that the booster would need to be administered yearly to combat the variants; however, the percent of healthcare workers who would take the yearly booster varied dramatically, depending on if they received the initial vaccine (45). This could have effects on the general public, especially if there is a growing trend of healthcare workers recognizing that there is a need for the vaccine but still are not willing to get it. As healthcare workers are a model in terms of good health practices, the decrease in vaccine hesitancy in this group may have contributed to the increased uptake in the greater population. Emphasizing prominent civilian groups and their response to the COVID-19 vaccine has been a strategy in the COVID-19 vaccine campaign and looping healthcare professionals in this category can provide additional success (46).

## 4. Booster hesitancy and acceptance abroad

Vaccine and booster hesitancy is seen abroad just like it is in the US. However, the rates of vaccine hesitancy and the primary reasons why people abstain from receiving the booster differ between countries. The COVID-19 vaccine booster dose is a relatively new concept and analyzing reasons for vaccine hesitancy during the first two doses can allow for utilization of more effective campaign strategies. It can reasonably be assumed that the same reasons for hesitation for the initial vaccination are still present for the decision of the booster dose. Approaches to addressing vaccine concerns should be individualized for each country and analysis of reasons why people are hesitant to receive the vaccine could aid in creating an individualized plan.

Table 1 shows different countries and the results of various studies of why people chose or did not choose to get the COVID-19 vaccine/booster in that country. These countries were chosen to sample each region of the world according to continent. They were also chosen to demonstrate the differences in reasoning for countries which have theoretically higher acceptance rates and those with lower acceptance rates according to a systematic review done in 2021 (63). The study measured the percent of participants intended to get the COVID-19 vaccine. Though vaccine acceptance rates have changed since then- at that time, Malaysia (94.3%) and China (91.3%) boasted some of the highest vaccine acceptance rates while Jordan (28.4%) and Italy (53.7%) had lower hypothesized vaccine uptakes. Japan and Greece were in the moderate range (63). Other countries such as Brazil and Australia are added to comprehensively evaluate a country from each continent.

**Table 1:** A sample of different countries and the primary reason why citizens there choose to get vaccinated or not get vaccinated.

**Country**	**% vaccinated (5)**	**% boosted (5)**	**Primary reasons to get vaccine**	**Primary reasons for vaccine hesitancy**
Japan (47)	79.94%	43.21%	1. Worrying about getting infected (33.2%) 2. Desire to protect family and friends (33.4%) 3. Societal pressure (31.7%)	1. Adverse reactions (73.9%) 2. Doubts about effectiveness of vaccine (19.4%)
Greece (48, 49)	73.27%	54.63%	1. Fear of contracting severe COVID-19 infection 2. Restrictions for social activities	1. Concerns about safety of vaccine (65.5%) 2. Doubts about effectiveness of vaccine (15.7%) 3. Pandemic is associated with hidden political agenda 4. Belief that COVID-19 doesn't pose a threat
Italy (50, 51)	79.24%	64.57%	1. Trust in safety of vaccine (63.2%) 2. Vaccines are an effective tool, individually and for community (44.8%) 3. No negative personal experiences with prior vaccinations (35%) 4. Trust in doctors and healthcare professionals (33.7%)	1. Not enough information on utility and safety of vaccine 2. Trust in scientific community
China (52–54)	85.91%	46.48%	1. Doctor's recommendation 2. Protecting friends and family 3. Social benefits	1. Concerns about vaccine safety 2. Vaccine quality and effectiveness 3. Perceived low risk of COVID-19
Jordan (41, 55, 56)	43.40%	6.21%	1. Fear of family members being infected with COVID-19 2. Death from COVID-19 3. Becoming infected with SARS-CoV2	1. Low confidence in healthcare 2. Belief that vaccine causes infertility, contains tracking device, and alters one's genes
Malaysia (57–59)	78.82%	48.40%	1. Effectiveness of vaccine to stop spread of COVID-19 2. Suggestions from the Ministry of Health	1. Concerns about side effects (95.8%) 2. Low confidence in vaccine safety (84.7%) 3. Lack of available information (80.9%) 4. Perceived low risk of COVID-19 5. Theories of vaccine read on social media
Brazil (60, 61)	76.82%	41.60%	No studies regarding the reasons of vaccine uptake in Brazil	1. Fear of adverse outcomes from the vaccine and thus being unable to fulfill daily responsibilities 2. Concerns of vaccine safety
Australia (62)	84.03%	53.84%	No studies regarding the reasons of vaccine uptake in Australia	1. Lack of information regarding vaccine 2. Uncertainty about safety of vaccine 3. Low perceived risk of COVID-19

Note that these statistics are before the roll-out of the bivalent COVID-19 booster.

Since the study and the release of the vaccine, China's vaccination rates have proved to be high at 90.20% for fully vaccinated citizens and 57.21% for boosted citizens (5). This is significantly higher than the rates of the US (80.67 and 39.89%) (5). The top reasons for intending to get vaccinated in China are physician recommendation and protecting friends and family. This sheds some light on the role of cultural norms in healthcare decisions. In China, emphasizing the vaccine/booster's ability to protect loved ones could encourage more citizens to get the COVID-19 vaccine as this is a bigger incentive than personal safety according to the results of the survey. If physicians were to discuss the vaccine with patients, it could contribute to increasing the vaccination rate as their insight is highly respected.

In Malaysia, though the current vaccination rate is not as high as the proposed vaccine acceptance rate was (82.85 and 49.77% for the booster), it still ranks higher than the global average and the US. However, low confidence in the vaccine and mistrust in the government were two of the top reasons that Malaysians chose to not get the vaccine. The implementation of a failed government program likely contributed to the discrepancy in anticipated vaccine hesitancy and actual vaccination rates (57). In February 2021, the Malaysian government proposed the creation of the NIP (National Immunization Program) which aimed to vaccinate 80% of the population by February 2022. Unfortunately in implementation, there was slow roll out of the vaccine and many faced obstacles in attaining the vaccine such as long wait times, logistical difficulties at the vaccination site, and lack of technological competence (64). This led to mistrust in the government which further lent itself to doubts in the effectiveness of the vaccine due to the lack of transparency surrounding the dissemination of the vaccine. COVID-19 vaccinations in Malaysia are a unique case study as the percent of people who were willing to get the vaccine was higher than the percent that received it.

Interestingly, over time, UK, US, Canada, and some European countries saw a drop in vaccine acceptance rates (63). This comes as a surprise as it would be expected that as the vaccine spends more time on the market, vaccine confidence would grow among the population. Government trust was cited as the most important factor in vaccine acceptance in these regions as well which emphasizes the important role of the public perception to the government as multiple countries are seen to be affected by this.

The low vaccine acceptance rate in Italy and Jordan was primarily owing to lack of safety information surrounding the vaccine and a low trust in the medical community (50, 51, 55). This reason is greatly seen in other Arab countries as well. A unique challenge to Jordan was fighting conspiracy theories and misinformation which had spread regarding the COVID-19 vaccine and booster. The anti-vaccination campaign promoted messages such as tracking devices inside the vaccine, DNA altering substances, and more. Given that the mRNA technology was new and many were already frustrated with the government's response to the pandemic, Jordanians were increasingly vulnerable to the anti-vaccination campaigns (55).

Cultural values and circumstances vary between different countries and therefore, reasons for vaccine hesitancy differ between countries. Looking at country specific reasons for vaccine and booster willingness and hesitancy provides evidence that messages advocating the COVID-19 vaccine should be targeted to the specific population it is addressing. In collectivist countries, like Jordan and China, emphasizing the benefits to family and friends might boost vaccination rates while greater transparency about the vaccine and its side effects might prove more effective in Italy. One limitation is that within a country, there are many different areas and provinces with different demographics. Thus, messages may have to be even more specifically tailored to the region for maximum effectiveness. This is exemplified in the vaccine uptake demographics of South Africa. Though each region in South Africa experiences some feelings of doubts about the vaccine side effects and effectiveness, the extent to which these feelings dominate the vaccine climate is dependent on the area of South Africa. Seventy-nine percent of citizens in South Africa who live in a city were willing to try a new vaccine while only 69% in villages were ready to try it (65). South Africa is reported as one of the “most unequal” countries in the world, meaning that income levels are extremely polarized. While regionalization plays a role in COVID-19 vaccine uptake in most countries, it is most evident in a country such as South Africa with wide income differences (66).

Even within countries of seemingly similar demographics, vaccine hesitancy reasons may differ. The same study which looked at the vaccination status of Americans in relation to their willingness to receive the booster also compared those statistics for participants from the UK. They found that more participants in the UK would be willing to receive the vaccine- regardless of if they had received the vaccine or not (67). This may be due to how the government responded to obstacles regarding the vaccine. When it was announced that the AstraZeneca vaccine in the UK and the Johnson & Johnson vaccine in the US posed a danger for blood clots, the US immediately pulled Johnson & Johnson from the market, before putting it back, while the UK adjusted its requirements to suggest that patients with greater risk of blood clots take the Pfizer or Moderna vaccine (68, 69). Policy like this may cause the American public to be fearful and cautious of the US COVID-19 policy. Furthermore, different regions in the same country have a large variation in population so a province-based analysis would be more useful than a country wide analysis, proving to be a limitation in our analysis (70).

## 5. Ethical discussion of COVID-19 boosters

There is no denying that COVID-19 boosters add an extra layer of immunity and are beneficial in decreasing COVID-19 disease severity. However, studies have already emphasized the global vaccine inequity between high-income and low-income nations. In September 2021, only 0.28% of the world's distributed COVID-19 vaccine doses have been in low-income countries (71). To date, only 28.31% of people in low income countries has received at least one dose whereas 72.8% in high income countries has received at least one dose (72). Offering booster doses in wealthier nations may serve to widen that gap.

Low and middle income countries (LMIC) have had higher mortality rates and transmission rates during the pandemic due to limited protective equipment, insufficient medical resources, and increased comorbid conditions (73). The COVID-19 vaccine offered relief from the consequences of the pandemic, but the dispersion of the vaccine has followed income lines with the poorest populations around the world having been unable to protect themselves with the vaccine. According to the World Health Organization (WHO), efforts to contain the pandemic would require 40% of people in every country to have been vaccinated by the end of 2021 and at least 70% vaccinated by June of 2022 (74, 75). Unfortunately, by the end of 2021, there were 98 countries which did not meet this goal and the majority are in Sub-Saharan Africa with vaccination rates around 10–20% (76, 77). According to projected coverage maps, by June of 2022, only high income and upper middle-income countries will meet the 70% benchmark. Low middle income countries will hover around a 65% mark while low-income countries will lag behind at 13%. By September 2022, as predicted- high income and upper middle income countries passes the 70% benchmark. Lower middle income countries were at 63% and low income countries had the least people vaccinated at 22% (78). Interestingly, low middle income countries were lower than their projection but low income countries performed better than the projection, though still significantly below the benchmark (projection was 13% but achieved 22% by September). This points to improvements in the dispersal of vaccines in LMIC but there changes are still required to achieve vaccine equity.

Since then, COVAX, a global vaccine sharing program, was started with the aim to increase vaccination coverage; unfortunately, it quickly fell behind its goal. This was largely attributed to slow funding, the need for more vaccine manufacturers, and blockages in shipping (77). Even if the doses were distributed more equitably around the globe, their short shelf life and lack of accessibility of citizens would pose additional challenges to lower income countries.

The onset of the booster campaign meant that higher income countries would allocate more vaccines for their citizens which would once again limit the availability of these vaccines for LMICs. If 11 of the richest countries were to provide booster vaccinations for citizens over 50 years of age, it would use ~440 million doses of the global supply (79). Wealthy nations have already begun large scale booster campaigns for anyone, regardless of age, so it is reasonable to assume that more than 440 million doses have been used as boosters. Vaccination of LMICs should be prioritized and greater focus should be given to strategies to increase the vaccination rate worldwide. This would not only be for the benefit of the currently underserved populations in LMIC. Such a shift in the vaccine paradigm would have a long-lasting positive impact on higher income countries as well. SARS-CoV-2 mutations are more likely to occur with higher rates of transmission; LMIC with low vaccination rate will continue to serve as hotspots for SARS-CoV-2 mutations that can quickly spread globally. We have already seen a decrease in effectiveness of the COVID-19 vaccine and further mutations could eventually render the vaccine ineffective (61). The spread of the Omicron variant has been documented to be in part due to global vaccine inequality; higher transmission rates in South Africa led to mutations that in turn spread world-wide (7, 80). The poorest countries will likely need to wait until 2023 before they are able to start offering vaccines to most of their population (81). This may lead to additional mutations as seen with the Omicron variant. Aside from moral considerations, high income countries also have a financial incentive to aid in the global vaccination campaign. For every $1 that high income countries spend on supplying vaccines to LMICs, they will see a return of $4.80 from raw material and goods that come from those countries (71). An increase in COVID-19 infection rates in LMICs means that the supply of raw materials will decrease, thus decreasing production and economies worldwide (71).

Even though the effectiveness of the vaccine has been decreasing with mutants, they are still very effective in preventing serious complications, hospitalizations, and death (82). When administering booster doses, countries stand to gain more from vaccination of the completely unvaccinated. While both are beneficial, the development of strategies to increase vaccination abroad, instead of increasing booster doses at home, may have more of a direct impact on the progress of the pandemic.

## 6. COVID-19 vaccine/booster mandates

As the COVID-19 pandemic evolves, there is a possibility of recurrent boosters and booster mandates (83). The benefits of mandating vaccination can logically be seen in terms of decreasing COVID-19 related health risks. However, there could be consequences to mandatory vaccinations as well.

Mandatory COVID-19 vaccination has already been put into effect by certain businesses and counties in the US. This is enforced by requiring people to present vaccine certification at entry (84). However, the implementation of mandatory vaccination remains a controversial dilemma and the future of the COVID-19 vaccine requirement is unclear. Abroad, some countries have already implemented mandatory COVID-19 vaccination and these countries can be used as a model to predict the result of this mandate in the US. A study looked at the increase in vaccination once the mandate was put into effect and found that vaccination rates increased 20 days before the implementation of the mandate and this increase in vaccination lasted for up to 40 days after the mandate was placed, specifically in France and Israel, where the pre-mandate vaccination rates were lower than average. However, the increase in vaccine uptake was not seen equally through different age groups. Those under 20 and 20–29-year-olds had the largest response to the mandate as vaccination rates in this age group increased the most. The most responsive age group also depended on what venues were only available to vaccinated people. For example, when nightclubs were restricted in Switzerland, increased vaccination rates was steepest with people under 20 years and it wasn't until more locations were restricted (any location with > 30 people) that vaccination rates increased significantly for other age groups (85). Mandated vaccination could lead to increased vaccine distribution in the US and possibly lead the country to the desired increased vaccination rate (86).

The development and recommendation of a booster dose brings up a greater urgency to settle the matter of mandatory vaccination. Some schools and businesses are requiring the booster dose; however, the CDC currently does not require that individuals take the booster to be considered “fully vaccinated” (87). This definition can create confusion and hesitancy for the encouragement of vaccine mandates. If the US decides to pursue mandatory vaccination, will this only include the initial vaccine doses? Additionally, if the initial two dose series is seen to have decreasing effectiveness over time, is there any benefit to requiring mandatory vaccination without the booster doses? Additional studies of the effectiveness of the COVID-19 vaccine over time will become more important in the decision to require mandatory vaccination or not.

Requiring the COVID-19 vaccine and booster among healthcare workers may have far reaching implications- both at an individual level and societal. There is little doubt that during the pandemic, occupational hazards for healthcare workers were high as they faced a greater exposure to the virus from their patients. Healthcare workers not only have a high risk of contracting the virus from their patient but also subsequently passing the contracted virus to future patients, many of which may have other health challenges making them prone to severe COVID-19 outcomes. By invoking the Hippocratic Oath, a physician's first duty is to “do no harm” to their patient. Mandating the COVID-19 vaccine would ensure that the physician is limiting the risk of COVID-19 transmission to their patient and taking every precaution possible to decrease this risk (88). As we have seen from the influenza vaccine, mandatory vaccination policies among healthcare workers was the most effective way to obtain maximum vaccination rates and minimizing the spread of influenza (89).

Citing the 4 main ethical principles in medicine brings forth an argument against mandatory vaccinations. The concept of patient autonomy has been one to guide medical practice for centuries. While relatively rare, side effects to the COVID-19 vaccine have been seen in a select few patients. Acute myocardial infarction, myocarditis/pericarditis, pulmonary embolism, and stroke have all been reported as adverse events for the vaccine (90). Patients should be informed about the risks as well as be able to make their own decisions regarding the cost and benefit of receiving the vaccine.

Furthermore, as previously discussed, the COVID-19 vaccine being the fastest vaccine to ever have been developed adds to the ethical dilemma of mandatory vaccination. No long term data about the safety of the vaccine/production has come out yet and part of the concern of mandating vaccines could be this gap in certainty. However, this will be a point of debate which doesn't dissipate. According to Morens et al., SARS-CoV-2 is unlikely to be eliminated and a growing need for a universal COVID-19 vaccine is becoming imperative to have broader immunity. Until then, zoonotic coronaviruses can continue to pose a threat and cause periodic outbreaks and endemics (91). In the meantime, until a broader vaccine is created, ongoing booster shots may be necessary to prevent a surge in coronavirus cases. Interestingly, studies have found that there is no difference in willingness to take the COVID-19 vaccine whether it is annual or not (28). However, using data from the influenza virus, vaccination from the flu has increased over the years (92). If the COVID-19 vaccine follows trend, there could be an increase in vaccination rate over time if a dose is needed yearly.

A primary reason for vaccine hesitancy is the lack of safety data, clinical studies, and knowledge about the vaccine. If the vaccine were required yearly, patients might feel more comfortable receiving the vaccine since it has been approved for a longer time. The CDC reports that in 1980, there were only 12.4 million doses of the influenza vaccine administered but by 2020, had increased to 194 million doses. Amount of doses given year to year differ but the overall trend is a strongly increasing trend (92). It can be assumed that over time, the COVID-19 vaccine will follow the same trend and higher vaccination rates will be achieved the longer that the vaccine is on the market and if the vaccine is mandated yearly.

## 7. Conclusion

It has been 2 years since the global spread of the SARS-CoV-2 virus, but the effects of the pandemic are still being felt around the world. The development of COVID-19 vaccines allowed for a chance to curb the viral spread and maintain a sense of normalcy but vaccine hesitancy and mutations in the spike protein limited the effectiveness of the vaccine. This led to the development of a successful booster dose schedule. Despite the health benefits that vaccines offer, the worldwide vaccination goal has not been reached and vaccine hesitancy to both the first vaccine doses and the booster are widespread. Hesitations for the vaccine include decreased vaccine literacy and scientific misinformation, side effects, and mistrust in the governments/pharmaceutical companies. The primary reasons for vaccine hesitancy differ between countries and analysis of the specific reason in each country can allow for a more targeted vaccine uptake. Countries such as China have high vaccine uptake rates and reasons for this include incentive to protect families and follow physician recommendations. On the other hand, countries like Jordan struggle with government mistrust and misinformation surrounding COVID-19 vaccines which have limited vaccine and booster rates. Additionally, there remains ethical concerns surrounding vaccine mandates and patient autonomy. Finally, the implementation of booster doses by high-income countries poses an additional challenge to ongoing vaccine inequity. Low resource settings have had difficulty in accessing primary COVID-19 vaccination; administration of widespread booster doses in resource rich countries could further the shortage of vaccines for communities in LMIC. As new technologies and policies are being built around the COVID-19 vaccine, continuing to monitor the effectiveness and public perception will prove vital for the future impact of COVID-19 as well as vaccines to come.

## Author contributions

AS: writing—original draft preparation, data collection—literature review, and data analysis and synthesis. OC: writing—review and editing. Both authors have read and agreed to the published version of the manuscript.
